# Genome-Wide Analysis of Interchromosomal Interaction Probabilities Reveals Chained Translocations and Overrepresentation of Translocation Breakpoints in Genes in a Cutaneous T-Cell Lymphoma Cell Line

**DOI:** 10.3389/fonc.2018.00183

**Published:** 2018-05-30

**Authors:** Anne Steininger, Grit Ebert, Benjamin V. Becker, Chalid Assaf, Markus Möbs, Christian A. Schmidt, Piotr Grabarczyk, Lars R. Jensen, Grzegorz K. Przybylski, Matthias Port, Andreas W. Kuss, Reinhard Ullmann

**Affiliations:** ^1^Max Planck Institute for Molecular Genetics, Berlin, Germany; ^2^Bundeswehr Institute of Radiobiology Affiliated to the University of Ulm, Munich, Germany; ^3^Department of Dermatology and Venerology, Helios Klinikum Krefeld, Krefeld, Germany; ^4^Berlin Institute of Health, Institute of Pathology, Charité – Universitätsmedizin Berlin, Corporate Member of Freie Universität Berlin, Humboldt-Universität zu Berlin, Berlin, Germany; ^5^Clinic for Internal Medicine C, University Medicine Greifswald, Greifswald, Germany; ^6^Human Molecular Genetics, Department of Functional Genomics, University Medicine Greifswald, Greifswald, Germany; ^7^Institute of Human Genetics, Polish Academy of Sciences, Poznan, Poland

**Keywords:** chromosome conformation capture, chromoplexy, chromosomal translocations, deep sequencing, cutaneous T-cell lymphoma

## Abstract

In classical models of tumorigenesis, the accumulation of tumor promoting chromosomal aberrations is described as a gradual process. Next-generation sequencing-based methods have recently revealed complex patterns of chromosomal aberrations, which are beyond explanation by these classical models of karyotypic evolution of tumor genomes. Thus, the term chromothripsis has been introduced to describe a phenomenon, where temporarily and spatially confined genomic instability results in dramatic chromosomal rearrangements limited to segments of one or a few chromosomes. Simultaneously arising and misrepaired DNA double-strand breaks are also the cause of another phenomenon called chromoplexy, which is characterized by the presence of chained translocations and interlinking deletion bridges involving several chromosomes. In this study, we demonstrate the genome-wide identification of chromosomal translocations based on the analysis of translocation-associated changes in spatial proximities of chromosome territories on the example of the cutaneous T-cell lymphoma cell line Se-Ax. We have used alterations of intra- and interchromosomal interaction probabilities as detected by genome-wide chromosome conformation capture (Hi-C) to infer the presence of translocations and to fine-map their breakpoints. The outcome of this analysis was subsequently compared to datasets on DNA copy number alterations and gene expression. The presence of chained translocations within the Se-Ax genome, partly connected by intervening deletion bridges, indicates a role of chromoplexy in the etiology of this cutaneous T-cell lymphoma. Notably, translocation breakpoints were significantly overrepresented in genes, which highlight gene-associated biological processes like transcription or other gene characteristics as a possible cause of the observed complex rearrangements. Given the relevance of chromosomal aberrations for basic and translational research, genome-wide high-resolution analysis of structural chromosomal aberrations will gain increasing importance.

## Introduction

The analysis of structural chromosomal aberrations is of relevance for both basic and translational research. Several chromosomal markers are already routinely used in clinical tests for genotype-based sub-classification of tumors or to assist in therapeutic decisions. In addition, the identification of recurrent aberrations can highlight driver genes of tumorigenesis, which represent promising starting points for the development of targeted therapies. Apart from clinical applications, the characterization of chromosomal aberrations can shed light on the underlying mutational mechanisms and in this way contribute to a better understanding of the cause and course of intra-individual evolution of tumors.

**Figure 1:**
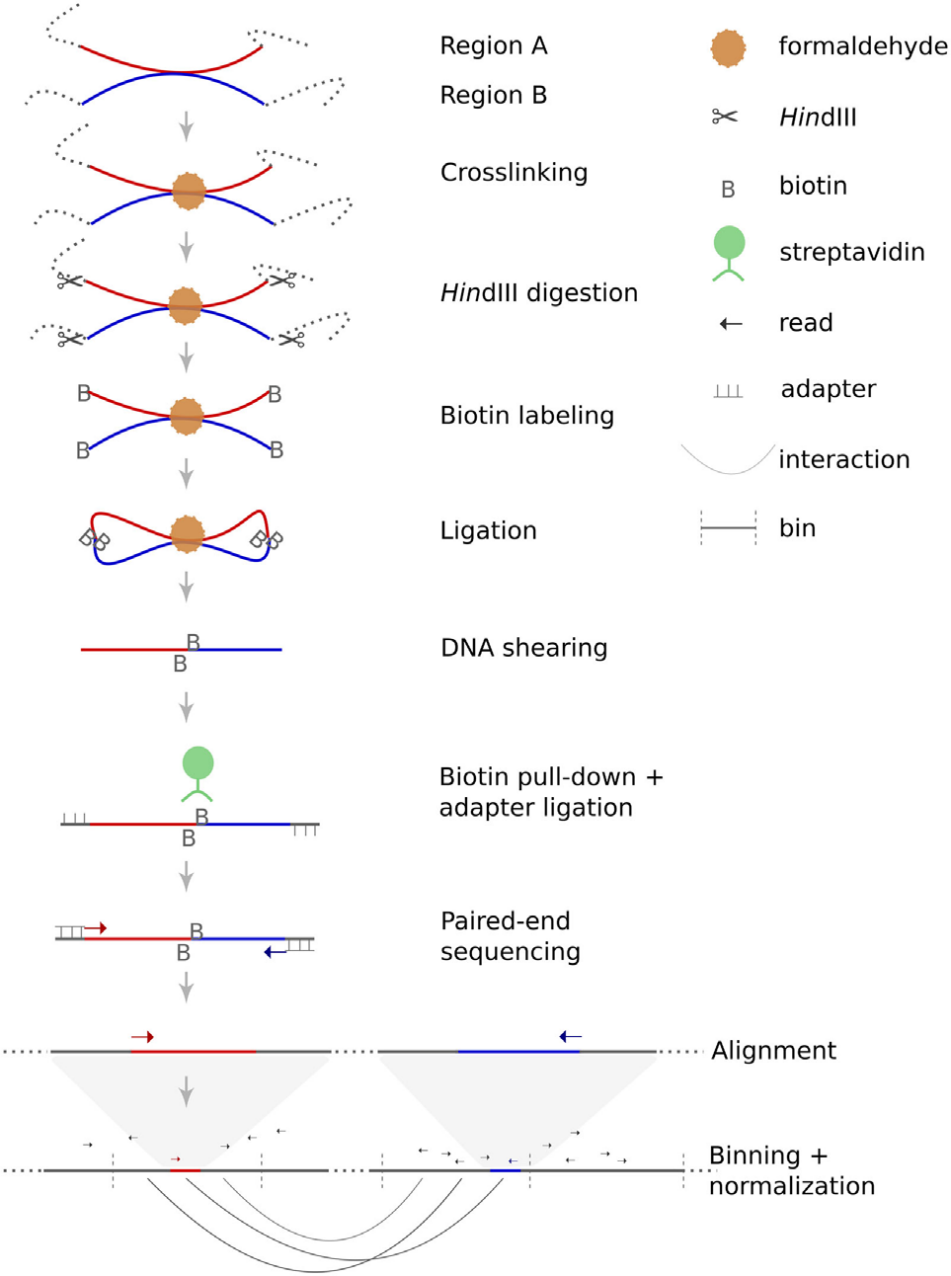
Principle of Hi-C. Hi-C is a variant of the chromosome conformation capture assay dedicated to the identification of genome-wide intrachromosomal and interchromosomal interaction probabilities. The method starts with crosslinking of chromatin within the nuclei. Restriction enzyme digestion (in this case HindIII) generates sticky ends which are filled-in and labeled with biotin. During the following ligation step, free DNA ends are re-ligated, which can either restore the original DNA sequence or can lead to chimeric products. The likelihood of such chimeric products depends on their spatial proximity. Afterward, crosslinking is reversed and DNA is sheared. Size selection and pull-down of biotin-labeled fragments are performed to increase specificity. Next, sequencing adapters are ligated to each fragment before sequencing. Finally, paired-end sequences are mapped to the human reference genome to deduce spatial proximities from the frequency of chimeric sequences.

According to classical models of tumorigenesis, complex abnormal karyotypes emerge through the stepwise acquisition of chromosomal rearrangements followed by expansion of those mutated clones with highest proliferative capacity (1). Yet, the conception of intra-individual karyotypic evolution has been biased by the limited perspective as provided by the low resolution of genome-wide datasets on structural chromosomal rearrangements for a long time. The lack of appropriate techniques capable of capturing karyotypic complexity both genome-wide and with high resolution has hampered the identification of mechanisms alternative to the well-documented gradual process. The introduction of array-based comparative genomic hybridization [(arrayCGH) (2, 3)] has mitigated this technical shortcoming for unbalanced structural chromosomal aberrations, but the situation has remained unsatisfactory for balanced chromosomal rearrangements. Until recently, their characterization required time-consuming cloning of breakpoints or, in case of translocations, depended on sophisticated sorting of derivative chromosomes followed by hybridization of sorted chromosomes on DNA microarrays (4–6). This situation has changed with the advent of next-generation sequencing (NGS), which has set the stage for the development of new protocols for the analysis of structural chromosome aberrations (7). Initially, these analyses have mainly focused on alterations of sequencing depth across the genome or along sorted chromosomes to define DNA copy number changes and translocation breakpoints (8), respectively. Later protocols have taken advantage of paired-end reads and used their mapping position and orientation with respect to the human reference genome to infer the presence and location of structural chromosome aberrations (9). Despite the development of paired-end NGS protocols, the identification of structural chromosomal aberrations such as balanced translocations has remained challenging. This is mainly due to the fact that strategies based on standard paired-end sequencing protocols have to rely on those few sequenced chimeric fragments that span the chromosomal breakpoints. Hence, reliable detection of such rearrangements using standard NGS protocols requires considerable sequencing depth (10). Furthermore, even in case of sufficient sequencing depth, translocation breakpoints in the very vicinity of regions with low mappability, such as repetitive elements, segmental duplications or DNA segments with extreme bias of base composition might be missed or erroneously aligned (11). An alternative strategy capable to overcome these problems is based on the fact that chromosomal rearrangements such as translocations disrupt nuclear architecture and modify spatial proximities of chromosome territories. These modifications of nuclear organization can be monitored by chromosome conformation capture assays such as Hi-C (12–14). This technique combines proximity ligation and NGS to infer nuclear neighborhood of chromosomal regions (see Figure 1 for explanation). The closer two chromosomal segments are within the nucleus, the more frequent Hi-C will detect interactions between them.

In general, interaction frequencies between two regions on the same chromosome decrease with linear distance and interactions between two chromosomes are considerably rare when compared to intra-chromosomal ones. In case of a translocation, regions of two or more chromosomes come into close contact. This results in an abrupt increase of interaction frequencies between the segments adjacent to the translocation breakpoints and makes Hi-C an ideal approach for the detection of balanced translocations (10, 15–17).

The application of array- and sequencing-based approaches as described above unveiled an unprecedented complexity of structural chromosomal aberrations in tumor genomes. In several cases, the observed mutational patterns were hardly compatible with a stepwise accumulation of chromosomal aberrations as described by the classical model of tumorigenesis (18). For example, regionally confined clusters of numerous chromosomal aberrations with limited DNA copy number states in the absence of general genomic instability have suggested a single catastrophic event as the underlying cause of this complex pattern of aberrations instead of a series of consecutive events. Meanwhile, it has been shown that this phenomenon, termed chromothripsis, can be encountered in a broad range of tumor types, where it can affect 2–3% of patients (19). In 2015, Zhang and colleagues succeeded to demonstrate that micronuclei formation and DNA damage confined to these structures can produce similar patterns of chromosomal changes as typical for chromothripsis (20). Multiple simultaneously arising DNA double-strand breaks also account for the emergence of chromoplexy, which is characterized by chained translocations and interlinking deletion bridges involving numerous chromosomes (21). The complex patterns of chromosomal aberrations typical for chromoplexy are unlikely result of a stepwise process, as this would require the repeated use of the same chromosomal breakpoints (18, 21). It is still unclear what triggers these simultaneously arising DNA strand breaks in the context of chromoplexy (18).

In this study, we have employed chromosomal interaction probabilities to fine-map translocations in a cell line derived from a patient with Sézary syndrome. The etiology of this highly malignant cutaneous T-cell lymphoma is poorly understood (22) and thorough investigation of structural chromosomal aberrations promises more insights into its development and progression (23). We demonstrate the presence of chained translocations partly connected by interlinking deletion bridges, which suggests the manifestation of chromoplexy and argues against a gradual appearance of these chromosomal aberrations. The overrepresentation of chromosomal translocation breakpoints within genes highlights the possible impact of spatial proximities of genes and biological processes associated with genes on the emergence of chromosomal translocations in this cutaneous T-cell lymphoma.

## Materials and Methods

Hi-C is a high-throughput variant of the chromosome conformation capture assay and facilitates the genome-wide investigation of interaction probabilities of genomic segments within the nucleus. The technology is based on crosslinking of chromatin, fragmentation of DNA followed by re-ligation. Depending on their spatial distribution, not only the original DNA fragments but also fragments in spatial proximity will re-ligate. These chimeric fragments can be detected by paired-end sequencing and their frequency can be used to calculate the interaction probability of these fragments within the nucleus. The principle of Hi-C is schematically depicted in Figure 1. For this study, we have used a Hi-C protocol published in detail by Lieberman-Aiden and colleagues (13).

### Fixation, Cell Lysis, and Restriction Enzyme Digestion

In brief, 20–25 million Se-Ax cells (24) were cross-linked with formaldehyde (Thermo Fisher Scientific, Waltham, MA, USA). Crosslinking was stopped by addition of 125 mM glycine (Merck Millipore, Darmstadt, Germany). After washing cells in ice-cold DPBS buffer (Lonza, Basel, Switzerland), cell pellets were flash frozen and stored at −80°C. For lysis, cells were resuspended in Hi-C lysis buffer and lysed using a Dounce homogenizer (Fisher Scientific GmbH, Schwerte, Germany). After centrifugation, pellets were washed twice in NEB buffer 2 (New England Biolabs, Ipswhich, MA, USA), finally resuspended in 370 µl NEB buffer 2 and 50 µl were transferred to seven tubes each. In order to remove proteins not cross-linked to DNA, 38 µl 1%SDS (Sigma-Aldrich, St. Louis, MO, USA) was added to each tube and incubated for 10 min at 65°C. Afterward, SDS was inactivated by the addition of 44 µl 10% Triton X-100 (Sigma-Aldrich, St. Louis, MO, USA). In each but one tube 400 U HindIII (New England Biolabs, Ipswhich, MA, USA) were added and DNA was digested overnight at 37°C with rotation. The next day, the tube with undigested DNA and one HindIII treated sample were removed to verify HindIII digestion efficiency.

### Endlabeling, Re-Ligation, Reversal of Crosslinking, and DNA Purification

For the remaining tubes a fill-in reaction was performed to blunt the sticky ends as generated by HindIII digestion. For later enrichment of re-ligated fragments, Biotin-dCTP (Invitrogen, Carlsbad, CA, USA) was incorporated during this fill-in reaction. Fragments were re-ligated with 15 U T4 DNA ligase per tube for 4 h at 16°C. Reversal of crosslinking was made by addition of 25 µl Proteinase K (20 mg/ml, Thermo Fisher Scientific, Waltham, MA, USA) and incubation overnight at 65°C. After RNA digestion with 50 µl RNase A (10 mg/ml, Thermo Fisher Scientific, Waltham, MA, USA) for 45 min at 37°C, DNA was purified by means of standard phenol chroroform isoamylalcohol treatment (Sigma-Aldrich, St. Louis, MO, USA) and ethanol (Merck Millipore, Darmstadt, Germany) precipitation. DNA of the separate tubes was conflated and concentration measured with a Qubit fluorometric assay (Thermo Fisher Scientific, Waltham, MA, USA).

### Removal of Biotin From Unligated DNA Ends, Enrichment of Re-Ligated Fragments and Preparation of Sequencing Libraries

In order to remove biotin-labeling from unligated fragments, samples were treated for 2 h at 12°C with 5 U T4 DNA polymerase (New England Biolabs, Ipswhich, MA, USA), whose exonuclease activity removed the biotin at the ends of the unligated fragments while keeping the centrally positioned biotin of ligated fragments untouched. Library generation was done according to the manufacturer’s protocols, with minor adaptions concerning the pull-down of biotin-labeled fragments to eliminate unligated fragments. In brief, 5 µg DNA was sheared with the Covaris S2 system (Covaris, Woburn, MA, USA), DNA end-repaired and size selected by means of Agencourt AMPure XP Reagent beads (Beckman Coulter Genomics, Danvers, MA, USA). Afterward, size and quantity was verified employing a 2100 Bioanalyzer (Agilent Technologies, Santa Clara, CA, USA). Unligated fragments were depleted by pull-down of biotin-labeled fragments with 50 µl streptavidin beads (10 mg/ml, Life Technologies, Carlsbad, CA, USA). The resulting DNA-coated beads were resuspended in 34 µl TE buffer (Life Technologies, Carlsbad, CA, USA). After A-tailing, i.e., addition of a dATP to the repaired DNA ends, and ligation of sequencing adapters, 5 µl of DNA-coated beads were used for 10 cycles of PCR amplification with primers complementary to the ligated sequencing adapters. These amplicons were sequenced using the SOLiD 5500X1 Sequencing Instrument (Life Technologies, Carlsbad, CA, USA) using the paired-end protocol with 75 and 35 nucleotides for the forward and reverse strand, respectively.

### Processing and Quality Control of Hi-C Data

Forward and reverse sequence reads were separately aligned to the human reference genome (hg19) by means of LifeScope Genomic Analysis Software 2.5.1. The resulting file was imported into the software tool HOMER v.4.7 (25), where the dataset was filtered for possible PCR artifacts, reads with low mapping quality and reads derived from sites lacking HindIII motifs. For visualization of chromosomal interaction probabilities as heatmaps, reads that passed the above-mentioned filters were summarized to genomic bins of 100 and 250 kb in size and read counts were normalized. The normalization strategy as implemented in HOMER proceeds on the assumption that each region within the genome should have the same visibility and for that reason equalizes possible artifactual effects caused by differences in GC content, accessibility of DNA and unequal distribution of HindIII sites. Significantly interacting genomic bins were determined employing HOMER’s analyzeHiC module (FDR = 0.001; bin interaction distance >25 Mb). Visualization of interaction frequencies in heatmaps was done in JAVA Treeview (26). Translocations were preselected by visual inspection of the interaction heatmaps. Translocation breakpoints were fine-mapped by evaluating read distribution within a 2 Mb window surrounding the breakpoints in order to identify the HindIII fragment next to the breakpoint.

### Comparison of Translocation Breakpoint Regions With Data on Higher Order Chromatin Conformation, DNA Copy Number Alterations, and Search for Expressed Fusion Genes

Given the hypothesis that spatial proximity might promote the emergence of translocations, we have processed public Hi-C data on the B-lymphocyte cell line GM12878 (27) in the same ways as the data for Se-Ax and evaluated the presence of significant interchromosomal interactions as defined by HOMER (FDR = 0.001) connecting the translocation partner chromosomes by means of Circos (28). Additionally, we have visually inspected various public data on chromatin interaction deposited at the 4DGenome database (29) (https://4dgenome.research.chop.edu/) to identify possible interactions between our intervals of interest in other cell lines.

Data on DNA copy number alterations in Se-Ax that have been generated by means of arrayCGH in a previous study (30) were visualized for each translocation within a 2 Mb interval surrounding the breakpoint by means of R and the R packages reshape2 (31) and ggplot2 (32). The expression of fusion genes was tested using previously published RNA-Seq data (33, 34). Translocation breakpoints were verified by screening paired-end sequencing data for Se-Ax (33, 34) and the analysis of these data by Breakdancer (35).

### Analysis of Chromosomal Breakpoint Overrepresentation Within Genes

Overrepresentation of translocation breakpoints within genes was tested at the resolution of single HindIII fragments by calculating the likelihood that the same number of randomly distributed HindIII fragments map to genes as it has been observed for HindIII fragments located next to the translocation breakpoints. In a first step, we have cataloged all HindIII restriction sites within the human genome by means of Galaxy Emboss command *fuzznuc* (36, 37). Gaps in the human genome assembly (38) were subtracted with BEDtools (39). From the resulting dataset, the Unix command *shuf* was employed to generate 100,000 permutations of 32 HindIII fragments and the BEDtools command “intersectBed” was used to compute the frequency of overlap with RefSeq genes (40).

To calculate the *p*-value for Monte Carlo resampling according to Ref. (41), the number of permutation datasets that feature an equal or greater count of HindIII fragment regions with gene overlap as observed (> = 24) were used as the expected overlap.

## Results

**Figure 2:**
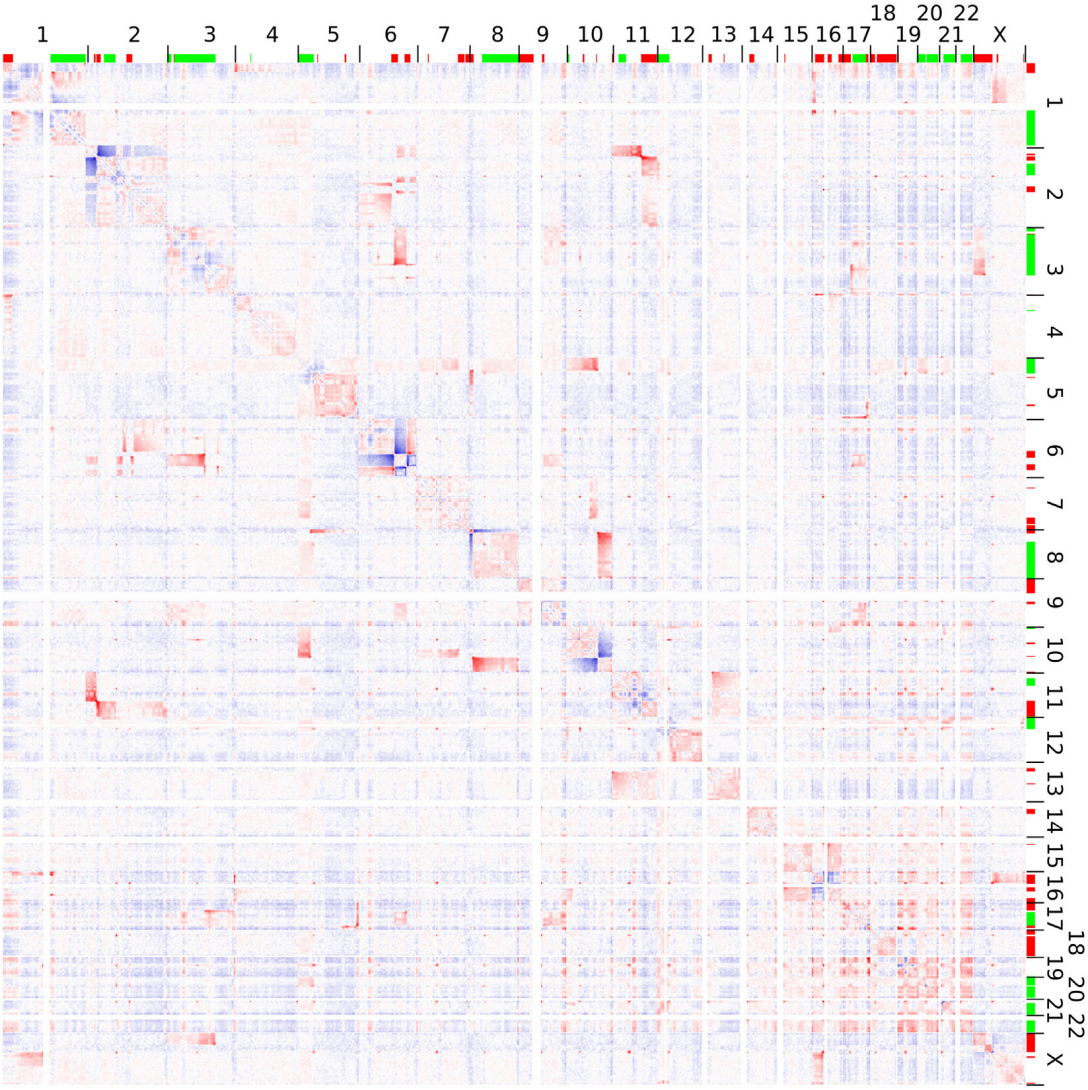
Genome-wide interaction frequencies in Se-Ax. Higher and lower than expected normalized interaction frequencies are shown with 2.5 Mb resolution in red and blue, respectively. The chromosome numbers are given at the top and to the right; together with information on DNA copy number losses (red) and gains (green) as detected by array comparative genomic hybridization. Translocations are characterized by interchromosomal interactions higher than expected, while their corresponding intrachromosomal interactions are decreased. A more detailed view of selected chromosomes is provided in Figure 3.

Hi-C analysis was based on 91.9 million read pairs that passed processing and quality filtering in HOMER. A genome-wide survey of structural aberrations is presented in Figure 2. This heatmap depicts the ratio of observed interaction frequencies and the expected frequencies based on a background model. Translocations are indicated by higher than expected frequencies of interchromosomal interactions (red color). Correspondingly, intrachromosomal interaction frequencies of the chromosomes involved in the translocation are decreased (blue color). The color gradient indicates the orientation of the breakpoint; i.e., interaction intensities decrease with distance from chromosomal breakpoints. In total, we identified 22 translocations, from which we were able to fine-map 32 breakpoints to a single HindIII fragment (Table 1; note that only 32 of the 34 breakpoints as listed in Table 1 were considered for the following analysis as in two cases breakpoints mapped to the same HindIII fragment). A comparison of Hi-C data with whole-genome sequencing data generated by a different laboratory using a different batch of Se-Ax cells (33, 34) revealed an overlap of 25 breakpoints. These have been highlighted in Table 1. A comparison of translocation breakpoints with array CGH data generated in a previous study by our laboratory with a resolution of ~100 kb (30) revealed that 11 of those breakpoints not identified by whole-genome sequencing were flanked by either deletions (*n* = 7) or duplications (*n* = 4). Other translocation breakpoints solely identified by Hi-C analysis were in close vicinity to other translocations, suggesting the presence of a complex rearrangement (t1/t10; t7/t8; t8/t15; and t13/t14). Yet, it has to be emphasized that non-overlapping breakpoints may also be owed to private mutations emerging during cultivation of Se-Ax cells in different laboratories over longer time or other technical reasons, in particular differences in resolution.

**Table 1:** Translocation breakpoints (hg19).

ID	Partner ID	Chromosome	Start	Stop	Genes
t1	t1_1p35.3	chr1	28238215	28248287	RPA2
	t1_4p16.3	chr4	4050000^b^	4150000	BC042823

t2	t2_1q21.3	chr1	150769818	150781274	CTSK
	t2_16p13.13^a^	chr16	11004475	11019710	CIITA

t3	t3_2q14.2^a^	chr2	120270564	120285427	SCTR
	t3_6q16.1^a^	chr6	98127299	98143928	LOC101927314

t4	t4_2q22.1^a^	chr2	137227625	137228172	—
	t4_6q21^a^	chr6	114395987	114399043	HDAC2-AS2

t5	t5_11q14.3^a^	chr11	88350000^b^	88450000	GRM5
	t5_2p22.3^a^	chr2	32260000^b^	32360000	Several genes

t6	t6_11q14.3^a^	chr11	88350000^b^	88450000	GRM5
	t6_2p22.3^a^	chr2	32230000^b^	32330000	Several genes

t7	t7_3q13.2	chr3	112240000^b^	112340000	Several genes
	t7_6q21^a^	chr6	106400000^b^	106500000	—

t8	t8_3q13.2	chr3	113435703	113437137	NAA50
	t8_17q11.2	chr17	28807394	28808819	GOSR1

t9	t9_3q24^a^	chr3	143214724	143222670	SLC9A9
	t9_Xp21.1^a^	chrX	36597000	36600264	—

t10	t10_16p13.13	chr16	11954332	11955153	—
	t10_4p16.3	chr4	3315595	3322490	RGS12

t11	t11_5p13.2^a^	chr5	37046678	37051545	NIPBL
	t11_8p23.1^a^	chr8	10977335	10981039	XKR6

t12	t12_10q23.31^a^	chr10	91799056	91801084	—
	t12_5q13.2^a^	chr5	37046678	37051545	NIPBL

t13	t13_17q25.1	chr17	70991114	71006897	SLC39A11
	t13_5q31.3	chr5	143656972	143667413	KCTD16

t14	t14_5q35.2^a^	chr5	176114621	176120288	—
	t14_17q24.3^a^	chr17	70537801	70539618	LINC00673

t15	t15_6q24.2^a^	chr6	143380000^b^	143480000	AIG1
	t15_17q11.2^a^	chr17	28770000^b^	28870000	CPD, GOSR1

t16	t16_10q22.1	chr10	71038330	71050820	HK1
	t16_7q31.33	chr7	126890674	126899921	GRM8

t17	t17_10q23.33^a^	chr10	95298787	95314132	—
	t17_8p23.1^a^	chr8	10977335	10981039	XKR6

t18	t18_12p12.2	chr12	20833396	20843983	PDE3A
	t18_10p14	chr10	7970549	7976904	TAF3

t19	t19_13q12.3^a^	chr13	31132320	31134497	HMGB1
	t19_11p15.5^a^	chr11	1444072	1449221	BRSK2

t20	t20_12p12.3	chr12	18675961	18680225	PIK3C2G
	t20_Xq28	chrX	147292149	147300244	—

t21	t21_17q25.3^a^	chr17	75511241	75516019	—
	t21_19p13.3	chr19	3737154	3750893	TJP3

t22	t22_17q24.3	chr17	69185554	69188239	CASC17
	t22_9q21.13	chr9	74350000^b^	74600000	Several genes

*^a^Identified in an independent Se-Ax cell batch by whole-genome sequencing*.

*^b^Not mapped to a single HindIII interval*.

*Start and stop define the HindIII interval covering the breakpoint*.

**Figure 3:**
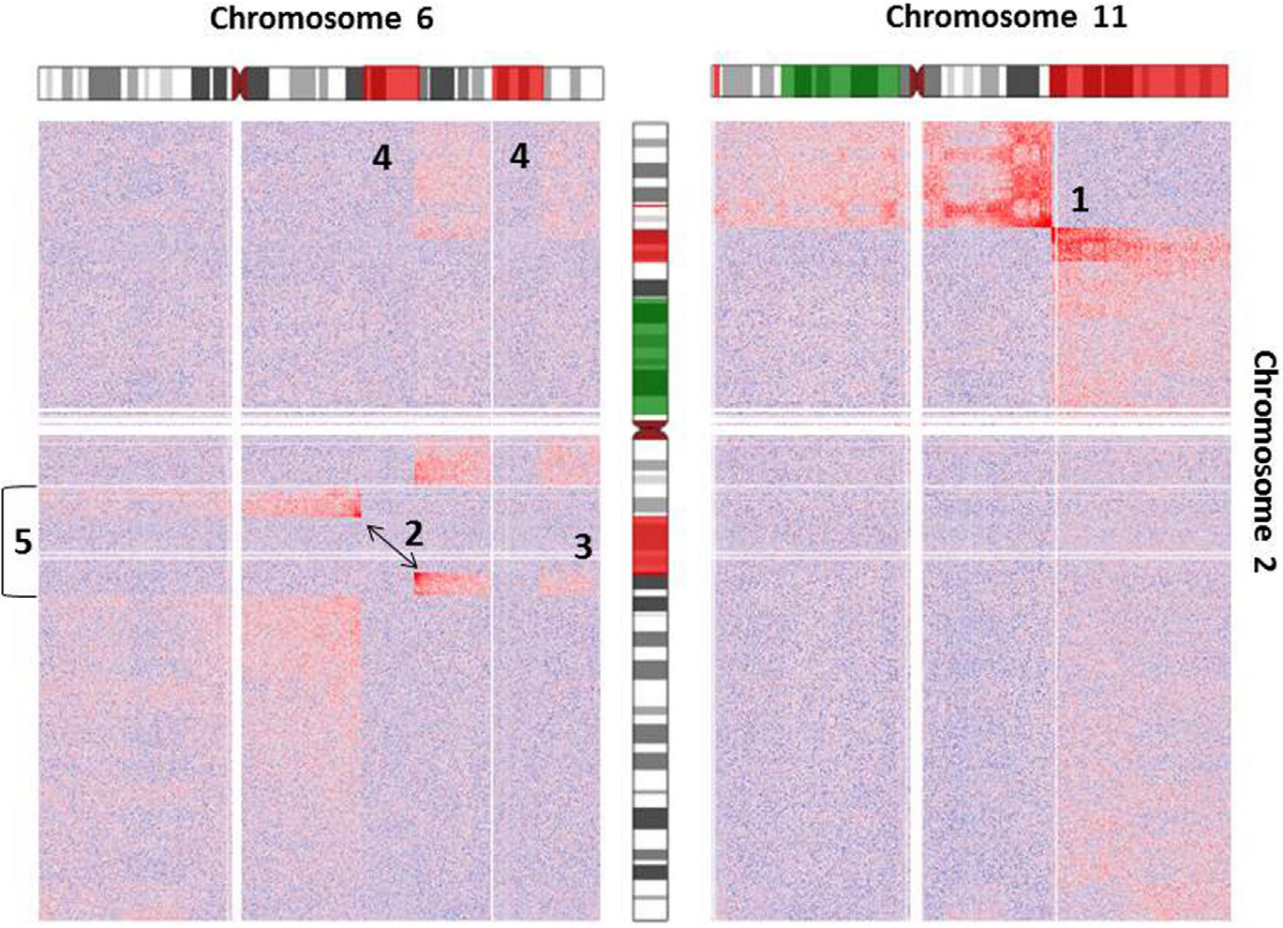
Heatmap of normalized interchromosomal interaction frequencies between chromosomes 2 and 6 and chromosomes 2 and 11. Two heatmaps are shown, which demonstrate the presence of a translocation t(2;6) (left) and t(2;11) (right), respectively. Both derivative chromosomes lead to higher than expected interchromosomal interaction frequencies, which are indicated by the red color gradient. Alterations of DNA copy number state as detected by array comparative genomic hybridization is indicated by coloring of the chromosome ideograms (red = deletion, green = gain). While the breakpoint of reciprocal translocation t(2;11) is easily identifiable [1], the identification of t(2;6) [2] is complicated by additional deletions of chromosome 2 [3] and chromosome 6 [4] and an inversion of chromosome 2 [5]. Orientation of chromosomal rearrangements can be inferred from the color gradient [interaction intensities (i.e., red color) decrease with distance from chromosomal breakpoints].

As an example for the complexity of chromosomal aberrations, a zoom-in depicting interchromosomal interactions for chromosomes 2, 6, and 11 is given in Figure 3. Additionally, chromosomal deletions and duplications identified by arrayCGH analysis of Se-Ax are indicated in both heatmaps.

**Figure 4:**
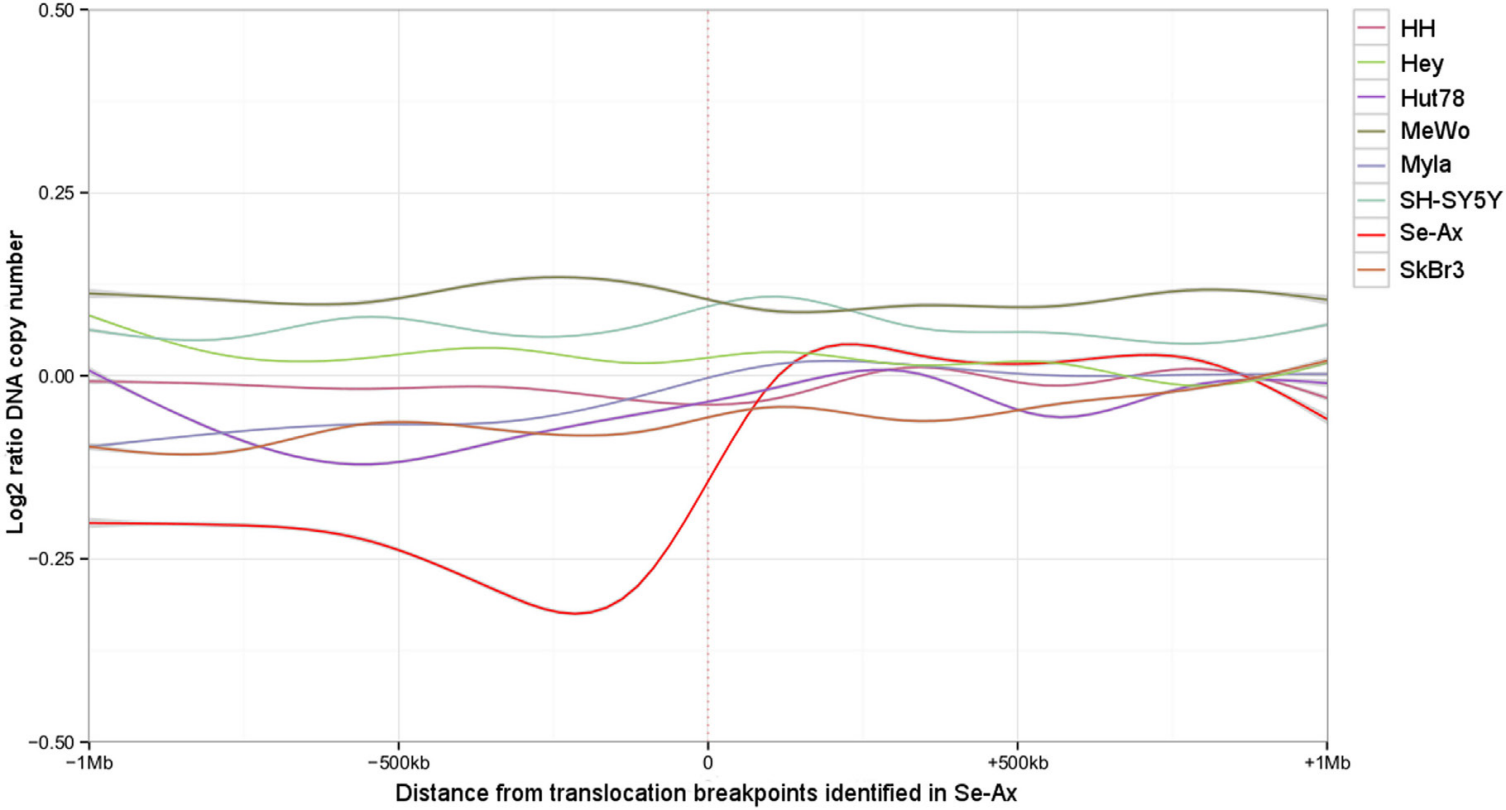
Deletions adjacent to the translocation breakpoints identified in Se-Ax. Smoothed log2 ratios of DNA copy number within a 2 Mb interval surrounding the translocation breakpoints are shown for Se-Ax (red line). DNA copy numbers of additional cell lines for the very same intervals are displayed for comparison (see insert box for color legend).

**Figure 5:**
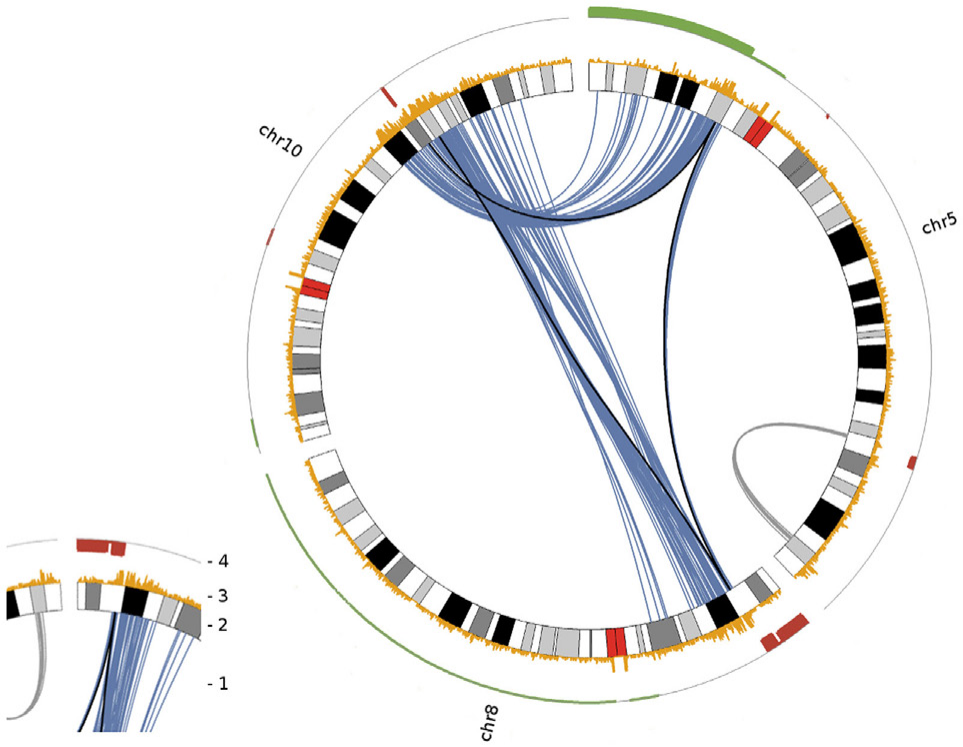
Circos plot visualizing chained translocations between chromosomes 5, 8 and 10. In this Circos plot chromosomes are radially aligned. Arcs within this circle indicate significant interchromosomal and long distance intrachromosomal interactions. Following the numbering given in the small insert to the left: (1) significant interchromosomal interactions (blue lines; FDR <0.001), significant long distance intrachromosomal interactions (gray lines; >25 Mb, FDR <0.001) and translocations as given in Table 1 (black lines); (2) radially aligned chromosome ideograms; (3) count of significant interactions per 50 kb bin (all interaction distances; max = 10); (4) DNA copy number status in red (deletion) and green (gain) as detected by array comparative genomic hybridization.

Deletions adjacent to transclocation breakpoints have been encountered 12 times (out of 32 breakpoints; Figure 4). The Circos plot depicted in Figure 5 demonstrates chained translocations with shared breakpoints between several chromosomes on the example of chromosome 5, 8, and 10.

In order to evaluate the impact of spatial proximity of chromosomes on the emergence of translocations, we screened public Hi-C datasets for interactions between those chromosomal intervals affected by translocations in Se-Ax. We failed to get any clues on higher interaction probabilities between regions encompassing the translocation regions, neither in the data of the lymphoblastoid cell line GM12878, which we processed the same way as the Se-Ax data, nor in the datasets from the 4DGenome database. Permutation analysis revealed a significant overrepresentation of translocation breakpoint-associated HindIII fragments within genes (*p* = 0.00208, 100,000 permutations). For one of the possible fusion genes (*AIG1/GOSR1*), transcripts were identified in the corresponding published RNA-Seq data (33).

## Discussion

The genome-wide identification and fine-mapping of chromosomal aberrations in tumor cells is instrumental in getting insights into the molecular mechanisms underlying their formation. On the example of a cutaneous T-cell lymphoma cell line we demonstrated the usefulness of Hi-C for the identification of balanced chromosomal translocations. We could show that the abrupt and prominent change of chromosomal interaction probabilities caused by derivative chromosomes facilitates the identification of translocation partners, their orientation to each other as well as their chromosomal breakpoints. Thereby, the observed changes of interaction probabilities were not confined to the area surrounding the breakpoints, but have extended several megabases beyond (Figures 2 and 3). This makes the detection of translocations by Hi-C sensitive, robust, and less prone to artifacts, particularly if the chromosomal breakpoints are next to repetitive sequences, segmental duplications or DNA copy number aberrations. Consequently, the analysis of translocations by Hi-C overcomes some of the limitations of alternative deep sequencing approaches described above. Resolution and sensitivity of this approach might be further increased by recent modifications of the Hi-C protocol (27), by using more sequencing reads and more frequently cutting enzymes (e.g., 4 bp instead of 6 bp) (42) or DNase as an alternative (43). A current limitation of the presented approach is that the presence of translocations has been identified by visual inspection of interaction matrices and that their chromosomal breakpoints were pinned down to the level of single restriction enzyme recognition sites by scrutinizing read distribution within the preselected chromosomal intervals later on. There is need for automation and objectivation of this process and very recently, first software tools dedicated to this task have already been presented (44). Although the focus of this study is on balanced translocations, the observation of higher than expected interaction probabilities between chromosomal segments can also be employed for the detection of DNA copy number alterations and inversions, but particularly for small inversions higher sequencing depth is needed for their robust detection (10). Hi-C analysis with sufficient sequencing depth and read length would also facilitate the determination of haplotype phase (45, 46), which would allow the correct assignment of chromosomal breakpoints to either the maternally or paternally derived chromosomes.

In line with previous reports, the Hi-C data presented in this study have revealed a highly complex karyotype in the investigated cell line. Strikingly, several of the translocation breakpoints seem to be chained and associated with chromosomal deletions. Such patterns of rearrangements have already been observed in other tumors (47, 48), including one case of cutaneous T-cell lymphoma (49), and the term chromoplexy has been coined to describe this phenomenon (21). Simultaneously arising DNA double-strand breaks are a prerequisite for chromoplexy, but the triggers of the temporarily and spatially confined genomic instability have not been identified yet (18). Notably, the majority of breakpoints map within genes (*p* = 0.00208), which suggests that transcription or other biological processes associated with genic sequences could be involved in this mutational event (50). Irrespective of the cause of genomic instability the fusion of DNA double-strand breaks requires their spatial proximity, either before DNA damage (contact first) or thereafter, when broken ends might migrate to some sort of repair center (breakage first) (51). Although our comparison of translocation breakpoints with public chromatin interaction data did not produce any conclusive results, there is evidence in the literature that nuclear neighborhood of chromosomes impacts the frequency of translocations (52, 53). Against this background, transcription factories could be the possible scene of the observed punctuated accumulation of chromosomal translocations, as genes from different chromosomes cluster together in these nuclear structures (54, 55).

In summary, we have demonstrated the power of genome-wide chromosome conformation capture analysis to detect chromosomal translocations. We present evidence that several translocations identified in the investigated cutaneous T-cell lymphoma cell line likely emerged simultaneously leading to karyotypic features typical of chromoplexy. Overrepresentation of breakpoints within genic sequences highlights the role of transcription or gene-associated biological processes in the emergence of the observed pattern of structural chromosomal rearrangements.

## Author Contributions

AS, GE, LRJ and AWK generated Hi-C data, which were analyzed by AS, GE and RU. GKP compared Hi-C results to whole genome sequencing data. CA, MM, GE, MP, CAS, BVB, PG LRJ, GKP and AWK contributed to data interpretation and critically reviewed the manuscript. AS and RU designed the study and wrote the manuscript.

## Conflict of Interest Statement

The authors declare that the research was conducted in the absence of any commercial or financial relationships that could be construed as a potential conflict of interest.
